# *Staphylococcus aureus* colonization of healthy military service members in the United States and Afghanistan

**DOI:** 10.1186/1471-2334-13-325

**Published:** 2013-07-16

**Authors:** Todd J Vento, Tatjana P Calvano, David W Cole, Katrin Mende, Elizabeth A Rini, Charla C Tully, Michael L Landrum, Wendy Zera, Charles H Guymon, Xin Yu, Miriam L Beckius, Kristelle A Cheatle, Clinton K Murray

**Affiliations:** 1 Brooke Army Medical Center/San Antonio Military Medical Center, Fort Sam Houston, 3551 Roger Brooke Drive, Fort Sam Houston, Texas 78234, TX, USA; 2 Infectious Disease Clinical Research Program, Uniformed Services University of the Health Sciences, Bethesda, MD, USA; 3 Blanch field Army Community Hospital, Fort Campbell, KY, USA; 4 United States Army Institute of Surgical Research, Fort Sam Houston, TX, USA

**Keywords:** Deployment, Malaria chemoprophylaxis, Doxycycline, Military, *Staphylococcus aureus*, Colonization

## Abstract

**Background:**

*Staphylococcus aureus* [methicillin-resistant and methicillin-susceptible (MRSA/MSSA)] is a leading cause of infections in military personnel, but there are limited data regarding baseline colonization of individuals while deployed. We conducted a pilot study to screen non-deployed and deployed healthy military service members for MRSA/MSSA colonization at various anatomic sites and assessed isolates for molecular differences.

**Methods:**

Colonization point-prevalence of 101 military personnel in the US and 100 in Afghanistan was determined by swabbing 7 anatomic sites. US-based individuals had received no antibiotics within 30 days, and Afghanistan-deployed personnel were taking doxycycline for malaria prophylaxis. Isolates underwent identification and testing for antimicrobial resistance, virulence factors, and pulsed-field type (PFT).

**Results:**

4 individuals in the US (4 isolates- 3 oropharynx, 1 perirectal) and 4 in Afghanistan (6 isolates- 2 oropharynx, 2 nare, 1 hand, 1 foot) were colonized with MRSA. Among US-based personnel, 3 had USA300 (1 PVL+) and 1 USA700. Among Afghanistan-based personnel, 1 had USA300 (PVL+), 1 USA800 and 2 USA1000. MSSA was present in 40 (71 isolates-25 oropharynx, 15 nare) of the US-based and 32 (65 isolates- 16 oropharynx, 24 nare) of the Afghanistan-based individuals. 56 (79%) US and 41(63%) Afghanistan-based individuals had MSSA isolates recovered from extra-nare sites. The most common MSSA PFTs were USA200 (9 isolates) in the US and USA800 (7 isolates) in Afghanistan. MRSA/MSSA isolates were susceptible to doxycycline in all but 3 personnel (1 US, 2 Afghanistan; all were MSSA isolates that carried *tetM*).

**Conclusion:**

MRSA and MSSA colonization of military personnel was not associated with deployment status or doxycycline exposure. Higher *S. aureus* oropharynx colonization rates were observed and may warrant changes in decolonization practices.

## Background

The US military has continually dealt with the challenges of controlling and treating *Staphylococcus aureus* infections, as was evident by the development of penicillin-resistant strains soon after penicillin’s introduction into battlefield medicine during World War II [1]. Increasing antimicrobial resistance remained a challenge during the Vietnam War and has continued to present-day in the wars in Iraq and Afghanistan [2-8]. In addition to the challenges with *S. aureus* in the war zone, methicillin-resistant *S. aureus* (MRSA) and methicillin-susceptible *S. aureus* (MSSA) have caused substantial skin and soft tissue infections among military personnel not deployed to a combat zone [9-11]. To mitigate excess morbidity and mortality from MRSA and MSSA, studies have attempted to better define pathogen epidemiology, identify associated risk factors for both colonization and infection; and implement decolonization strategies [12-14]. However, several studies suggest continuing epidemiological shifts in antimicrobial resistance profiles, sites of colonization, and infection rates of MRSA and MSSA [9,15-20].

Given the ongoing healthcare challenges of MRSA and MSSA in military personnel in austere environments, a better understanding of microbial epidemiology dynamics in the context of deployment is necessary to facilitate effective disease control strategies. Combat deployments, as well as humanitarian and disaster relief missions, often occur in environments not conducive to adequate hygiene practices. Further, these deployments are often to geographic regions that require antimalarial chemoprophylaxis. The use of doxycycline for malaria prevention has raised concerns about potential inducible resistance and its impact on MRSA treatment and decolonization [21]. Therefore, we conducted a pilot study of two populations of military personnel (one in the US and one in Afghanistan) to determine MRSA and MSSA colonization prevalence and evaluate different anatomic sites, pulsed-field types (PFTs), antimicrobial resistance, and *S. aureus* virulence and resistance genes.

## Methods

### Participants

Two populations of healthy active duty service members (101 non-deployed personnel in San Antonio, Texas, USA and 100 personnel deployed to Afghanistan) were assessed in this study. Recruitment of participants occurred in an outpatient medical clinic, at the time of presentation for acute, non-urgent care. All participants were 18 years or older. Exclusion criteria for the non-deployed participants were: overseas travel or deployment within 6 months, antibiotic use within 30 days, and an active infection that may have altered a study participant’s normal flora or involved a proposed anatomic sample site. The deployed personnel resided in a single province in Afghanistan for approximately 7 months. These were healthy individuals without active infections, who were taking doxycycline for malaria chemoprophylaxis (100 mg orally daily). Demographic characteristics were obtained using a short questionnaire. Brooke Army Medical Center and the US Army Medical Research and Material Command Institutional Review Boards approved the two studies and consent forms.

### Surveillance cultures

#### Troop medical clinic, San Antonio, TX

Cultures were collected from seven anatomical sites (nare, oropharynx, axilla, groin, web spaces of dominant hand, web spaces of the foot, and perirectal area) using pre-moistened swabs (Copan, Stuart liquid media culture, Copan Inc., Brescia, Italy) over a 2 month time period (May-June, 2011). Swabs were immediately transported to the laboratory and plated onto Trypticase Soy Agar with 5% sheep blood (sheep blood agar), as well as CHROMagar™ *S. aureus* plates for rapid detection of *S. aureus*. After incubation at 35°C, colonies with morphology consistent with *S. aureus* on sheep blood agar and mauve colored large colonies from CHROMagar™ *S. aureus* media were subcultured onto sheep blood agar in order to ensure culture purity. All isolates were frozen at −80°C in Trypticase Soy Broth (TSB) with 15% glycerol.

#### Acute care clinic in Afghanistan

Samples were collected from deployed participants in Afghanistan using BD CultureSwab^TM^ Max V(+) (Becton Dickinson, Franklin Lakes, NJ), which contain Amies medium and a unique blend of non-animal proteins embedded into the swab fibers, providing additional nutrients for survival of microorganisms during transport. Collection of samples occurred over a one month time period (August, 2011) using the same method described for the US study site. The isolates were stored at room temperature, as this was previously shown to be the optimal storage temperature for preservation and stability of collected bacteria for up to 4 weeks. The swabs were sent to San Antonio Military Medical Center in two separate shipments (14 days apart) for pathogen recovery as described above.

#### Antimicrobial susceptibility testing

Frozen isolates underwent automated testing for identification and susceptibilities with the BD Phoenix Automated Microbiology System (Becton Dickinson and Company, Franklin Lakes, NJ), using the PMIC/ID-107 panels per manufacturer’s instructions. Confirmation for doxycycline susceptibility of all *S. aureus* isolates was done using the PMIC/ID-106 panels. MRSA was defined as *S. aureus* with evidence of oxacillin or cefoxitin resistance and detection of the *mecA* gene. MSSA was defined as *S. aureus* without resistance to oxacillin or cefoxitin and no detection of the *mecA* gene.

#### Molecular testing

Frozen isolates of *S. aureus* underwent pulsed-field gel electrophoresis (PFGE) as described elsewhere [22]. PFGE gels were analyzed using the commercial software BioNumerics (Applied Maths Inc., Austin, TX) and clonality was assessed using established criteria [22,23]. Isolates were screened for the presence of antimicrobial resistance genes coding for resistance to various antimicrobial agents including β-lactam antibiotics (SCC*mec*), methicillin-oxacillin (*mecA*), macrolide-lincosamide-streptogramin (*ermA, ermB, ermC, ermT*), macrolide (*msrA*), penicillin (*blaZ*), tetracyclines (*tetK, tetL, tetM, tetO*), trimethoprim (*dfrA, dfrK*), aminoglycosides (*aac*(6′)*-aph*(2′)), quaternary ammonium compounds (*smr, qacA/B*), and mupirocin (*mupA*). Using PCR, isolates were also screened for the presence of virulence factors (Panton-Valentine leukocidin - PVL, Arginine Catabolic Mobile Element - ACME) and accessory gene regulators (Agr) associated with pathogenicity of the organisms.

For PCR testing, genomic DNA was extracted from overnight cultures using the QIAamp DNA Mini Kit (Qiagen, Valencia, CA) following the manufacturer’s protocol. All PCRs were conducted with Eppendorf Master Mix (Eppendorf, Hamburg, Germany) containing 1.25 U Taq DNA polymerase, 1.5 mM Mg2+ and 200 μM of each dNTP final concentrations in the PCR and with a final volume of 25 μl. The PVL gene was detected using 400 nM of primers *luk-PV-1*, 5′-ATCATTAGGTAAA TGTCTGGACATGATCCA-3′, and *luk-PV-2*, 5′-GCATCAACTGTATTGGATAGCAA AAGC-3′ amplifying a 433 bp PVL product and the following PCR conditions: 94°C for 2 min followed by 30 cycles of 30 s at 94°C, 30 s at 55°C and 1 min at 72°C followed by a final extension 5 min at 72°C. *S. aureus* ATCC 49775 was used as positive amplification controls. The isolates were screened for the presence of the *arcA* gene by PCR using the primer pair *arcA-F* 5′-GAGCCAGAAGTACGCGAG-3′ and *arcA-R* 5′-CACGTAACTTGCT AGAACGAG-3′. The *arc*A gene belongs to the *ar*c gene cluster which is a surrogate marker for type I ACME. Amplification was carried out for 30 cycles with denaturation at 94°C for 20 s, annealing at 55°C for 30 s, extension at 72°C for 30 s, and a final extension at 72°C for 5 min using 400 nM concentrations of the primers resulting in an amplification product of 724 bp. *S. epidermidis* ATCC 12228 was used as positive control for all PCR runs. All PCR products were resolved by electrophoresis through 1.2% or 2% agarose gels and visualized with ethidium bromide.

### Statistical analysis

All statistical analyses, to include descriptive statistics and categorical variable comparisons using Pearson χ-square or Fisher’s Exact Test, were performed using SPSS (IBM®SPSS®Statistics Version 19). All p values were two-tailed and a value of <0.05 was considered statistically significant.

## Results

### Troop medical clinic, San Antonio, TX

Of 101 participants, two refused collection at the perirectal site and one refused collection at the oropharyngeal site, resulting in a total of 704 collected swabs. Median age was 23 years (IQR 22, 23), with a male predominance (69%). Most (85%) of the individuals had been born in the United States and 81% were in the Army. Study participants had been on active duty military service for a mean time of 9 months and assigned to their current base for a mean period of 3 months.

### Acute care clinic, Afghanistan

Collection of samples from 100 healthy participants deployed to Afghanistan yielded 700 samples. Median age was 24 years (IQR 24, 25) and all study participants were male. All personnel were in the Army and 84% of them performed foot patrols outside of the base, possibly predisposing them to more local population and environmental exposures.

### Comparison of *Staphylococcus aureus* colonization in US and Afghanistan personnel

#### MRSA

There were 4 personnel colonized with MRSA in each study population (Table 1). All MRSA colonized personnel in Afghanistan participated in operations outside of the base. In the US population, MRSA was isolated from oropharynx and perirectal sites while the Afghanistan MRSA isolates were identified from oropharynx, nares, hand and foot swabs (Table 1). All MRSA isolates in the US personnel were isolated from a single site on each subject, while 2 Afghanistan-based personnel had co-colonization with the same strain of MRSA at different anatomical sites (one with nares and oropharynx and the other with hand and foot co-colonization). Of the US-based personnel, 3 (75%) participants were colonized with community-associated USA300 MRSA, and 1 (25%) with healthcare-associated USA700 MRSA. Comparison of USA300 and USA400 MRSA in the US and Afghanistan personnel revealed statistical significance of increased prevalence of USA300 isolates in the US personnel compared to the Afghanistan personnel (p < 0.01).

**Table 1:** **Differences in methicillin-resistant *Staphylococcus aureus *(MRSA) and methicillin-susceptible *S. aureus *(MSSA) colonization in 101 healthy US military personnel in the US and 100 US military personnel deployed to Afghanistan**

	**MRSA**			**MSSA**		
**Demographics**	**US**	**Afghan**	**Total**	**US**	**Afghan**	**T otal**
Number of personnel (isolates)	4 (4)	4 (6)	8 (10)	40 (71)	32 (65)	72 (136)
Age median (IQR)	24 (24,25)	23 (22,23)	23 (23,24)	21 (20,25)	23 (22,26)	22 (21,25)
Gender – males%	50	100	75	69	100	84
Operations off the military base^a^		4	4		25	25
**Location of isolate colonization**						
Nares		2	2	15	24	39
Oropharynx	3	2	5	25	16	41
Axilla			0	4	4	8
Groin			0	7	6	13
Hand		1	1	4	6	10
Foot		1	1	8	9	17
Perirectal	1		1	8		8
**Number of sites colonized by subject**^**b**^						
1	4	2	6	21	15	36
2		2	2	9	8	17
3			0	7	4	11
4			0	3	3	6
5			0	0	2	2
**Personnel co-colonized at multiple sites**						
Nares and oropharynx		1	1	2	6	8
Nares and hand				1		1
Nares and axilla					1	1
Nares and groin				2	1	3
Nares and perirectal				1		1
Hand and foot		1	1			
Oropharynx and perirectal				1		1
Oropharynx and foot				1		1
Groin and hand				1		1
Nares, oropharynx and groin				1	1	2
Nares, oropharynx and perirectal				1	1	2
Nares, axilla and perirectal				1		1
Nares, groin and foot				1	1	2
Nares, oropharynx and foot				1	1	2
Nares, oropharynx and hand				1	1	2
Axilla, groin, foot and perirectal				1		1
Nares, oropharynx, axilla and foot				1		1
Nares, oropharynx, groin and foot				1		1
Nares, hand, groin and foot					1	1
Nares, oropharynx, axilla and hand					1	1
Nares, oropharynx, hand and foot					1	1
Nares, oropharynx, axilla, hand and foot					1	1
Nares, oropharynx, axilla, groin and foot					1	1

^a^Only Afghanistan personnel eligible.

^b^p-value <0.01 – Comparing MSSA and MRSA sites of colonization in all study personnel.

All MRSA isolates, from both populations, were susceptible to doxycycline, minocycline, tetracycline, rifampin, and trimethoprim-sulfamethoxazole in addition to vancomycin and daptomycin. Half of the MRSA isolates in the US personnel and 100% of MRSA isolates in the Afghanistan personnel demonstrated fluoroquinolone susceptibility. Clindamycin resistance was noted in 25% of isolates, with the *ermC gene* found in 25% of MRSA isolates in US personnel. 25% of the US MRSA isolates appeared to have inducible clindamycin resistance, based on automated testing results for inducible MLS_b_ phenotype. Additional resistance genes tested, including *aac*(6′)*-aph*(2′) for aminoglycoside resistance, *smr*, *qacA/B*, and *mup,* were not found in any MRSA isolates.

Only 25% of all MRSA isolates in Afghanistan and US personnel possessed PVL, and 50% of US and 25% of Afghanistan MRSA isolates were positive for ACME. Agr I was found in 100% of US and 25% of Afghanistan isolates, and Agr II was found in 25% of Afghanistan isolates (Table 2).

**Table 2:** **Pulsed-field types, antimicrobial resistance, and resistance and virulence genes, of methicillin-resistant *S. aureus *(MRSA) and methicillin-susceptible *S. aureus *(MSSA) isolates from personnel screened in the US and Afghanistan**

	**MRSA**			**MSSA**		
	**US**	**Afghan**	**Total**	**US**	**Afghan**	**Total**
**Pulsed-field types **^**a**^	**4**	**4**	**8**	**42**	**35**	**77**
***Community-associated isolates n(%)***	3(75)	1(25)	4(50)	4(10)	3(9)	7 (9)
USA 300	3(75)^c^	1(25)	4^(^50)^d^	2(5)^c^	2(6)	4(5)^d^
USA 400	0	0	0	2(5)	1(3)	3(4)
***Healthcare-associated isolates n(%)***	1(25)	3(75)	4(50)	38(90)	32(91)	70(91)
USA 200	0	0	0	9(21)	6(17)	15(20)
USA 500	0	0	0	0	5(14)	5(6)
USA 600	0	0	0	2(5)	1(3)	3(4)
USA 700	1(25)	0	1(12)	0	2(6)	2(3)
USA 800	0	1(25)	1(12)	7(16)	7(20)	14(18)
USA 900	0	0	0	5(12)	2(6)	7(9)
USA 1000	0	2(50)	2(12)	2(5)	5(14)	7(9)
***Other (%)***	0	0	0	13(31)	4(11)	17(22)
***Antimicrobial susceptibility and presence of antimicrobial resistance gene n(%)***^**b**^	4	4	8	47	35	82
Ampicillin	0	0	0	0	0	0
Ampicillin-sulbactam	0	0	0^e^	47(100)	35(100)	82(100)^e^
Penicillin G	0	0	0	10(21)	6(17)	16(19)
*blaZ*	4(100)	4(100)	8(100)	36(77)	29(83)	65(79)
Clindamycin	3(75)	4(100)	7 (87)	39(83)	29(83)	68(83)
*ermA*	0	0	0	8(17)	4(11)	12(15)
*ermB* and *ermT*	0	0	0	0	0	0
*ermC*	1(25)	0(0)	1 (12)	0	0	0
Erythromycin	0	1(25)	1 (12)^f^	34(72)	22(63)	56(68)^f^
*msrA*	3(75)^g^	3(75)	6(75)^h^	2(4)^g^	8(23)	10(12)^h^
*ermA*	1(25)	0	1(12)	8(17)	4(11)	12(15)
Levofloxacin	2(50)^i^	4(100)	6(75)^j^	46(98)^i^	35(100)	81(99)^j^
Moxifloxacin	2(50)^k^	4(100)	6(75)^l^	46(98)^k^	35(100)	81(99)^l^
Rifampin	4(100)	4(100)	8(100)	47(100)	35(100)	82(100)
Doxycycline	4(100)	4(100)	8(100)	46(98)	33(94)	79 (98)
Minocycline	4(100)	4(100)	8(100)	47(100)	33(94)	80(98)
Tetracycline	4(100)	4(100)	8(100)	46(98)	25(71)	71(87)
*tetK*	0	0	0	0^m^	6(17)^m^	6(7)
*tetL* and *tetO*	0	0	0	0	0	0
*tetM*	0	0	0	1(2)^n^	2(6)^n^	3(4)
Trimethoprim - sulfamethoxazole	4(100)	4(100)	8(100)	45(96)	35(100)	80(98)
*dfrA* and *dfrK*	0	0	0	0	0	0
**Other resistance and virulence markers (%)**^**b**^	**4**	**4**	**8**	**47**	**35**	**82**
*mecA*	4(100)	4(100)	8(100)	0	0	0
SCC*mec*	4(100)	4(100)	8(100)	0	0	0
PVL	1(25)	1(25)	2(25)^o^	1(2)	1(3)	2(2)^o^
ACME	2(50)^p^	1(25)	3(37)^q^	0^p^	0	0^q^
Agr I	4(100)	1(25)	5(62)	15(32)	11(31)	26(32)
Agr II	0	1(25)	1(12)	15(32)	12(34)	27(33)
Agr III	0	0	0	0^r^	11(31)^r^	11(13)

^a^ Includes isolates with unique PFTs that were also unique to an individual study participant.

^b^ Includes isolates that were unique to individual study participants.

Statistically significant differences (p <0.05):

^c^USA 300 in MRSA vs. MSSA colonized personnel for the US personnel.

^d^USA 300 in MRSA vs. MSSA colonized personnel for the total personnel.

^e^Ampicillin-sulbactam susceptibility in MRSA vs. MSSA colonized personnel for the total personnel.

^f^Erythromycin susceptibility in MRSA vs. MSSA colonized personnel for the total personnel.

^g^msrA gene presence in MRSA vs. MSSA colonized personnel for the US personnel.

^h^msrA gene presence in MRSA vs. MSSA colonized personnel for the total personnel.

^i^Levofloxacin susceptibility in MRSA vs. MSSA colonized personnel for the US personnel.

^j^Levofloxacin susceptibility in MRSA vs. MSSA colonized personnel for the total personnel.

^k^Moxifloxacin susceptibility in MRSA vs. MSSA colonized personnel for the US personnel.

^l^Moxifloxacin susceptibility in MRSA vs. MSSA colonized personnel for the total personnel.

^m^tet*K* presence in MSSA colonized personnel for US vs. Afghan personnel.

^n^tet*M* presence in MSSA colonized personnel for US vs. Afghan personnel.

^o^PVL presence in MRSA vs. MSSA colonized personnel for the total personnel.

^p^ACME presence in MRSA vs. MSSA colonized personnel in US personnel.

^q^ACME presence in MRSA vs. MSSA colonized personnel in total personnel.

^r^Agr III presence in MSSA colonized personnel for US vs. Afghanistan personnel.

#### MSSA

A total of 136 MSSA isolates were found to colonize 72 individuals: 40 personnel stationed in the US and 32 deployed to Afghanistan (Table 1). The most commonly colonized site was the oropharynx (62%) in the US personnel and nares (50%) in the Afghanistan personnel, and most of the participants were colonized at a single anatomical site (Table 1). Two US-based individuals were co-colonized with MRSA and MSSA at different sites, while no Afghanistan-based individuals were colonized with both MSSA and MRSA. A total of 77 unique (by genotype and individual study participant) MSSA isolates (42 in the US and 35 in the Afghanistan personnel) were identified and are represented under the CA- and HA-MRSA sections in Table 2. Only 10% of the US and 9% of the Afghanistan MSSA isolates were found to be USA300 and USA400 types (most commonly associated with community-associated strains), while the remainder were other USA and non-USA strain-types (Table 2). The most common MSSA PFTs were USA200 (9 isolates) in the US and USA800 (7 isolates) in Afghanistan.

All isolates were susceptible to ampicillin-sulbactam and rifampin. Clindamycin susceptible isolates were found in 83% of personnel in each study group, with evidence of the presence of the *ermA* gene in 17% of the US and 11% of the Afghanistan personnel. Fluoroquinolone resistance (2%) was only seen in the US study group. Minocycline susceptibility (98% of total isolates, 100% in US study group) was greater than tetracycline susceptibility (87% of total MSSA isolates), and the *tetK* and *tetM* genes were identified in isolates from the Afghanistan study personnel (Table 2). 98% of US-based and 94% of Afghanistan-based isolates were doxycycline susceptible. Trimethoprim-sulfamethoxazole resistance of 4% was seen only in the US personnel.

Additional resistance genes tested were *aac*(6′)*-aph*(2′) for aminoglycoside resistance (0% in US and 48% Afghanistan isolates), *smr* (2% US isolates), and *qacA/B*, *mupA* (not found in any MSSA isolates). Only 2% of all MSSA isolates carried PVL, while none carried ACME. Agr I and Agr II were present in approximately one third of MSSA isolates (Table 2).

### Comparison of MRSA and MSSA isolates

PFT USA300 was common among MRSA isolates (75%) for the US-based personnel (Table 2). Ampicillin-sulbactam, erythromycin, and fluoroquinolone (levofloxacin and moxifloxacin) susceptibility was significantly more prevalent among MSSA isolates (Table 2). There were no differences in doxycycline, minocycline or tetracycline susceptibility between MRSA or MSSA isolates recovered in the US or Afghanistan. Significantly more MRSA isolates were associated with virulence factors such as PVL and ACME and the erythromycin resistance gene, *msrA*, whereas more MSSA isolates were associated with tetracycline resistance genes (*tetK, tetM*) (Table 2).

## Discussion

Continual surveillance for MRSA and MSSA colonization is required to ensure appropriate care is being provided, especially when people are located in austere environments, such as war or natural disasters, and exposed to antimicrobial pressure, such as antimalarial chemoprophylaxis. Earlier studies of healthy military personnel, carried out at the same US base, revealed MRSA and MSSA colonization rates of 1.5-4% and 20-38%, respectively; however, these studies only screened the nares [10,12]. In our study, six out of 8 MRSA colonized personnel would not have been detected if nare-only surveillance was performed and among MSSA isolates, colonization of the oropharynx was higher among personnel in the US than in Afghanistan. A study of VA patients in Boston indicated that only 2% of personnel had extra-nare only colonization; however, these patients did not have typical community-associated MRSA risk factors [18]. An urban emergency department (ED) study revealed 39% MSSA (156 of 400 personnel) and 5% MRSA (20 of 400 personnel) colonization [17]; 80% of personnel with MRSA were extra-nare and 45% were only extra-nare, with the oropharynx being the most commonly colonized site. A cross-sectional study of adults and children with *S. aureus* skin infections and their household contacts indicated that a nare-only survey would have missed 38% of MSSA and 51% of MRSA colonized persons [20]. Overall, most studies focus on patient populations with a history of ongoing skin infection or substantial risk factors of MRSA colonization; however, the literature increasingly supports the role of extra-nare site screening, especially the oropharynx [24-26].

As shown in previous studies of US military personnel, USA300 was a commonly recovered MRSA strain, accounting for only 55% of colonizing nare isolates, which is the same percentage as the urban ED colonization study [10,12,17]. The family transmission study showed USA300 to represent 16% of CA-MRSA and 16% of CA-MSSA isolates [20]. Data has suggested that extra-nare colonization might be associated with different rates of active infections; however, there is limited clinical data to support this assumption [27]. In our study, PVL was present in only 2 (25%) MRSA isolates and 2 (2%) MSSA isolates. This is in contrast to previous military colonizing studies showing nearly 50-67% of MRSA isolates having PVL [10,12]. Although the accessory gene regulator (Agr) was present in both study sites, the only statistical difference was Agr III’s presence in MSSA isolates from personnel in Afghanistan [28].

Despite the use of doxycycline antimalarial prophylaxis in the deployed population, there was no difference in doxycycline resistance between the US and Afghanistan personnel (for both MRSA and MSSA isolates). However, there was substantially more tetracycline resistance, especially in association with *tetK* resistance genes, in the isolates from Afghanistan personnel. The use of doxycycline did not appear to induce its own resistance (even in the presence of tet*K* resistance genes) as was shown in a prior study [21]. Our findings are also consistent with a national US survey of discordance between doxycycline susceptibility and tetracycline resistance [29]. Of 823 USA300 isolates, 72 (9%) were tetracycline resistant, and 69 of those were doxycycline susceptible [29]. In contrast, a prior French military study of personnel deployed to Cote d’Ivoire (and on doxycycline malaria chemoprophylaxis) described 2 outbreaks with PVL+, doxycycline-resistant MRSA infections [30]. Our findings are also supported by studies of tetracycline therapy for acne showing no increased rates of tetracycline resistance in MRSA or MSSA isolates, with possible decreased colonization rates [31].

The use of antimicrobial agents active against MRSA and MSSA could have a major impact on the care of casualties on the battlefield or those injured during natural disasters or humanitarian relief operations. Due to the high rate of infections associated with combat-related injuries, a standard practice is to initiate antimicrobial therapy at the time of injury [32-34]. Oral moxifloxacin is currently the recommended field antimicrobial for the US military unless there is an intraabdominal injury, for which ertapenem is recommended [33]. However, antimicrobial agents recommended by the International Committee of the Red Cross and British military are penicillin or aminopenicillins [33-35]. Once patients arrive to a US facility with surgical support, cefazolin is the recommended antimicrobial agent [33]. Studies have shown that wounds are colonized with MRSA and MSSA at the time of injury [36]. There is also data which supports that moxifloxacin does not select its own resistance for MRSA/MSSA as it occurs with other fluoroquinolones [37]. However, well controlled studies are needed to determine the role of post-injury antimicrobials and the most efficacious agents that also limit selection of increasingly resistant pathogens [38].

There are a number of potential limitations associated with this study. Although the two study populations were sampled with different swabs and specimens had different transportation/storage times prior to pathogen identification, prior studies (unpublished data by our group) have shown stability of MRSA/MSSA on different swab types at different temperatures and storage times. In addition, the similar colonization rates, colonization sites, and antimicrobial resistance between the MRSA/MSSA isolates collected in the US and Afghanistan make this unlikely an issue. The current study also did not use broth enrichment media, which may have decreased the diagnostic sensitivity of bacterial culture results. The current study determined point prevalence of MRSA/MSSA colonization and does not provide a complete understanding of the timeline of colonization in these populations. It is not clear whether the Afghanistan-based population data on overall colonization, MRSA/MSSA differences, or anatomic-site distribution differences reflect their exposure to the deployed environment or simply reflect their baseline colonization. Prospective incidence studies of military personnel prior to deployments and prior to malaria chemoprophylaxis/antibiotic exposure are needed to enhance understanding of *S. aureus* colonization in these populations. It is unclear if doxycycline use may have decreased MRSA and MSSA colonization prevalence from a level that would have been higher if agents without MRSA/MSSA activity (e.g. mefloquine, primaquine or atovaquone/proguanil) had been used for malaria chemoprophylaxis. Individual doxycycline compliance was not systematically assessed as part of this study which could contribute to an exposure misclassification; however, deployed unit healthcare providers estimated compliance to be very high, based on existing oversight practices for ensuring medication compliance in a combat zone. The study is also relatively small, limiting its power to detect actual differences in prevalence and resistance, and perhaps precluding the generalization of the findings to larger populations. On the other hand, the MRSA and MSSA colonization rates in this study are similar to previous studies conducted in military and civilian populations. Further, performing large clinical studies in a combat zone remains a significant logistical challenge.

## Conclusions

In this pilot study, MRSA and MSSA colonization rates were not substantially different based on deployment status and exposure to an austere environment where antimalarial agents with MRSA/MSSA activity are used. However, the finding of increased extra-nare site *S. aureus* (MRSA and MSSA) colonization should warrant further investigation, as many decolonization practices focus on nare and skin, but do not include the oropharynx. Prospective cohort studies are needed to determine the role of pre-injury colonization on the development of subsequent infections in deployed service members, and to enhance our understanding of the selection of resistant pathogens and the impact of decolonization and infection control measures in the deployed setting.

## Abbreviations

MRSA: methicillin-resistant *Staphylococcus aureus*; MSSA: methicillin-susceptible *Staphylococcus aureus*; PFT: pulsed-field type; PEGE: pulsed-field gel electrophoresis; PVL: Panton-Valentine leukocidin; ACME: Arginine Catabolic Mobile Element; Agr: accessory gene regulators; ED: emergency department.

## Competing interests

The authors declare that they have no competing interests.

## Authors’ contribution

Study design, Data Collection, Data Analysis, Manuscript Development, Writing Manuscript: TJV, TPC, DWC, KM, MLL, CKM. Data Collection, Manuscript Development: TPC, EAR, CCT, WCZ, CHC, XU, KAC, MLB. All authors read and approved the final manuscript.

## Pre-publication history

The pre-publication history for this paper can be accessed here:

http://www.biomedcentral.com/1471-2334/13/325/prepub

## Disclaimer

The views expressed herein are those of the authors and do not reflect the official policy or position of the Department of the Army, Department of the Air Force, Department of Defense, or the US Government. The authors are employees of the US government. This work was prepared as part of their official duties and, as such, there is no copyright to be transferred.

This work was supported by the Armed Forces Health Surveillance Center including the Global Emerging Infectious System.
